# Heat and Mass Transfer in an Adsorbed Natural Gas Storage System Filled with Monolithic Carbon Adsorbent during Circulating Gas Charging

**DOI:** 10.3390/nano11123274

**Published:** 2021-12-02

**Authors:** Evgeny M. Strizhenov, Sergey S. Chugaev, Ilya E. Men’shchikov, Andrey V. Shkolin, Anatoly A. Zherdev

**Affiliations:** 1 Research Institute of Power Engineering, Bauman Moscow State Technical University, ul. Baumanskaya 2-ya, 5, 105005 Moscow, Russia; chugaev@bmstu.ru (S.S.C.); i.menshchikov@gmail.com (I.E.M.); shkolin@bk.ru (A.V.S.); azherdev@bmstu.ru (A.A.Z.); 2 Frumkin Institute of Physical Chemistry and Electrochemistry, Russian Academy of Sciences, Leninskii Prospect, 31, bld. 4, 119071 Moscow, Russia

**Keywords:** adsorption, nanoporous carbon, monolithic adsorbent, methane storage, adsorbed natural gas, circulating charging, heat and mass transfer

## Abstract

Adsorbed natural gas (ANG) technology is a promising alternative to traditional compressed (CNG) and liquefied (LNG) natural gas systems. Nevertheless, the energy efficiency and storage capacity of an ANG system strongly depends on the thermal management of its inner volume because of significant heat effects occurring during adsorption/desorption processes. In the present work, a prototype of a circulating charging system for an ANG storage tank filled with a monolithic nanoporous carbon adsorbent was studied experimentally under isobaric conditions (0.5–3.5 MPa) at a constant volumetric flow rate (8–18 m^3^/h) or flow mode (Reynolds number at the adsorber inlet from 100,000 to 220,000). The study of the thermal state of the monolithic adsorbent layer and internal heat exchange processes during the circulating charging of an adsorbed natural gas storage system was carried out. The correlation between the gas flow mode, the dynamic gas flow temperature, and the heat transfer coefficient between the gas and adsorbent was determined. A one-dimensional mathematical model of the circulating low-temperature charging process was developed, the results of which correspond to the experimental measurements.

## 1. Introduction

Adsorbed natural gas (ANG) storage systems possess a number of significant advantages over compressed (CNG) and liquefied natural gas (LNG) technologies, in particular high energy efficiency caused by the lack of extremely high pressures and cryogenic temperatures as well as increased fire and explosion safety due to the “bound-state” of gas molecules with the surface of the adsorbent, the so-called “nano-dispersed state” [1]. In addition to safe storage tanks, ANG technology is characterized by other principles of organizing charging processes, which opens up opportunities for creating an energy efficient, safe, and economical charging infrastructure. However, it is well known that the efficiency of ANG systems strongly depends on significant thermal effects occurring during charge (adsorption) and discharge (desorption) processes, which requires the development of new approaches for the charging processes and thermal management of the ANG tank [2]. Another very important challenge for practical use of ANG is the development of new processes for “fast” charging of such systems.

As a rule, to reduce the influence of thermal effects special thermal management systems can be used: heat exchangers installed inside [3,4,5,6,7] or outside [8,9,10,11] the storage tank including the circulation of gas heated or cooled in an external heat exchanger [12,13,14,15]. In any thermal management systems, heat and mass transfer processes in the adsorber play a key role in ensuring efficient charging. Often in the scientific literature there are diagrams and dynamic temperature dependencies at different location points of the adsorber [6,12,16,17], but rarely are such data analyzed to obtain practical results. Due to its technical complexity, circulating charging is the least studied among other methods of thermal management.

There are also other approaches to deal with the thermal effects of adsorption/desorption processes. For example, in [18], a passive increase in the efficiency of the ANG system was studied by the method of mathematical modeling, namely, the effect of external convective heat exchange from the ambient air on internal thermal processes in an adsorption accumulator, depending on the external shape of the tank. The authors of this paper argue that this approach can reduce operating costs compared to active methods of increasing the efficiency of ANG systems, such as using internal or external heat exchangers. Zhao et al. [19] suggested using an aluminum alloy insert in the form of a honeycomb structure to increase the thermal conductivity of the adsorbent layer and tested this solution on an adsorber equipped with metal–organic framework MIL-101(Cr) with a low intrinsic thermal conductivity. The results showed that the temperature change during charging and discharging decreased by about 2 times, i.e., the heat-conducting insert is quite effective. Also, one of the effective methods for increasing the thermal conductivity of the adsorbent layer is the addition of expanded natural graphite (ENG) [20]. It was shown in [21] that the thermal conductivity of compacted activated carbon with the addition of ENG in comparison with the initial granular activated carbon was increased from 0.36 to 2.61 W·(m⋅K)^−1^. Prosniewski et al. [22] considered the issue of thermal effects in relation to the use of the ANG system on a car. The work is interesting, using a full-size adsorber with a honeycomb construction and a volume of 40 L. The authors proposed raising the charging pressure by a certain value as an option for “fast” charging, but noted that a small increase in pressure (about 1.0 MPa) provides within 3 min only about 80% of the capacity achievable with isothermal (“slow”) charging. For gas delivery, the authors proposed using forced gas deflation from the adsorber. The same authors analyzed the beneficial effect of thermoregulation on reducing the accumulation of impurities from natural gas in the adsorbent [23].

The main correlations for simulating the characteristics of an ANG storage system are presented in [24,25].

A circulating charging process with thermal management is considered as one of the most promising techniques regarding the implementation of “fast” charging of the ANG system with advanced efficiency. Therefore, this article is dedicated to the experimental study of a full-scale pilot circulating charging system with controlled thermal management for charging an ANG tank prototype filled with monolithic nanoporous carbon adsorbent.

## 2. Materials and Methods

### 2.1. Monolithic Carbon Adsorbent

Microporous (or nanoporous because of nanoscale pores) carbon adsorbents are the most promising materials today for application on ANG due to their high activity to methane, mechanical strength, and the possibility to increase packing density by shaping under pressure [26,27,28], which ensures the maximum volumetric storage capacity of the system.

**Figure 1:**
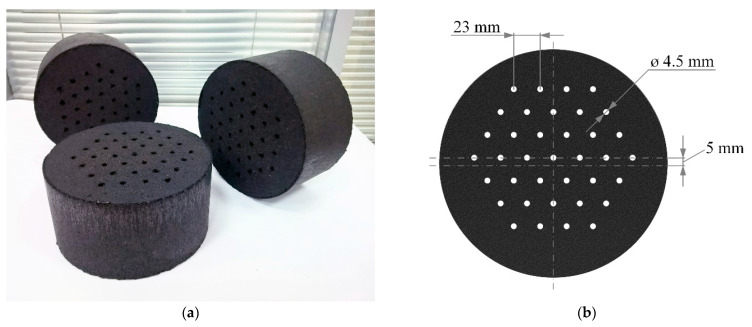
Samples of shaped monolithic carbon adsorbent AU-1 (**a**) and channels geometry of adsorbent monolith (**b**).

We used the industrial granulated activated carbon AU-1, which possessed a remarkable adsorption activity to methane and could potentially be used in ANG systems [29]. The AU-1 adsorbent has the following structural–energy characteristics: a specific micropore volume *W*_0_ = 0.62 cm^3^/g, a characteristic adsorption energy of benzene *E_0_* = 19.7 kJ/mol, and an average effective half-width of micropores *x*_0_ = 0.61 nm. The manufacturing technology of shaped cylindrical monoliths from AU-1 activated carbon is described in [30]. Compared to the original granulated adsorbent, the packing density of the shaped material was increased from 380 to 705 kg/m^3^. The average diameter and thickness of the monoliths were 196 and 101 mm, respectively. To reduce the hydraulic losses of the samples, 37 perforating channels with a diameter equal to 4.5 mm were made in the monoliths. Monolithic adsorbent samples (14 pcs.) were installed in an adsorption tank of 51 L inner volume. The estimated occupation of the inner space of the tank by the monoliths was about 80%. Figure 1a shows a photograph of samples of a monolith adsorbent before the installation in the adsorption tank. Figure 1b shows the geometry of the arrangement of channels in the monolith. The total surface area of the adsorbent monoliths was as follows: the outer cylindrical surface was 0.86 m^2^, the edge surface was 0.82 m^2^, and the surface of the channels was 0.72 m^2^. Such a number of holes play the role of a flow equalizer rather than increasing the heat transfer surface area.

The thermal conductivity of the manufactured adsorption monoliths was measured using the MIT-1 material thermal conductivity meter, which is designed to quickly determine the thermal conductivity of various materials by the probe method in accordance with the Russian State Standard GOST 30256-94 [31]. The principle of operation of the device is based on measuring the change in the temperature of the probe for a certain time when it is heated with constant power. The limits of the permissible relative error of the thermal conductivity meter are ±7%. As a result of several measurements, the average value of the thermal conductivity of the adsorbent monoliths was obtained, which was λ = 0.33 W·(m⋅K)^−1^. The obtained data on the thermal conductivity of monoliths are in good agreement with the data obtained from a similar active carbon in [32].

### 2.2. Adsorptive

Natural gas used in internal-combustion engines with technical specifications determined by the Russian State Standard GOST 27577-2000 [33] was used in the experiments. The composition of the natural gas was as follows, vol.: 96.1% methane, 2.2%; ethane, 0.8%; propane, 0.6%; and nitrogen and other impurities, less than 1%. Theoretical calculations and experimental data analysis were carried out on the basis of 100% (pure) methane. It had the following physicochemical characteristics [34]: molar mass μ = 16.043 g/mol, boiling temperature *T*_0_ = 111.67 K, critical temperature *T*_cr_ = 190.56 K, and critical pressure *p*_cr_ = 4.599 MPa.

### 2.3. Experimental Setup

**Figure 2:**
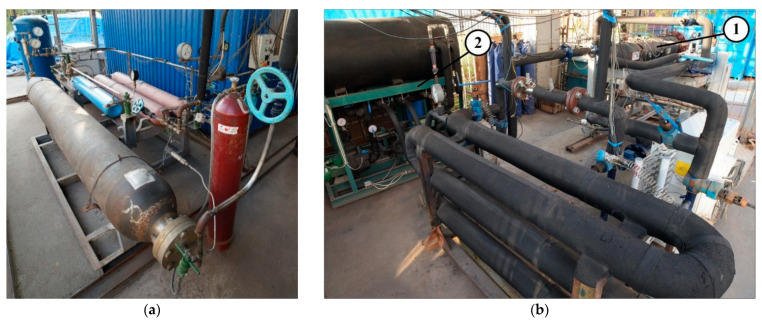
Experimental setup for study of natural gas charging and discharging processes from ANG storage system: (**a**) high pressure gas preparation unit and (**b**) circulating charging unit including adsorption tank (1) and coolant preparation unit (2).

To study the natural gas charging and discharging processes from the ANG storage system, a special multifunctional experimental setup was developed and manufactured. Photos of this setup are presented in Figure 2. The experimental setup has several operating modes: in agreement with the process—natural gas charging and discharging; in accordance with the heat transfer during charging—“adiabatic”, “isothermal”, and “low-temperature”; in accordance with the number of stages—single-stage and multi-stage charging [35]; and in agreement with the rate of the processes—“fast” and “slow” charging.

The experimental setup is designed to determine the macroscopic performance indicators for charging of various types, the optimal charging modes, and the time dependencies of various parameters. This setup also allows some indicators of heat and mass transfer to be determined.

The main dependencies for calculating the characteristics of ANG storage systems are presented in [24]. Charging without the removal of adsorption heat is the fastest charging method, but at the same time the least efficient in the amount of accumulated gas. Charging with the removal of adsorption heat to the environment takes longer but, due to a decrease in the temperature of the adsorbent, the amount of accumulated gas increases. Low-temperature charging [30] makes it possible to additionally cool the adsorbent after removing the heat of adsorption into the environment. Cooling during charging can significantly increase the amount of accumulated gas or significantly reduce the filling pressure [36]. With decreasing temperature, the proportion of “bound” gas in the ANG storage system increases, which provides increased fire and explosion safety of the storage system. This method allows, at a certain low temperature, the use of a natural gas compressor to be abandoned, which is one of the least reliable and at the same time the most expensive component of natural gas filling stations, and replaces it with a safe and reliable cooling unit. To cover the “peak” loads at the station, it is possible to use the supply of safe coolant instead of gas accumulators.

**Figure 3:**
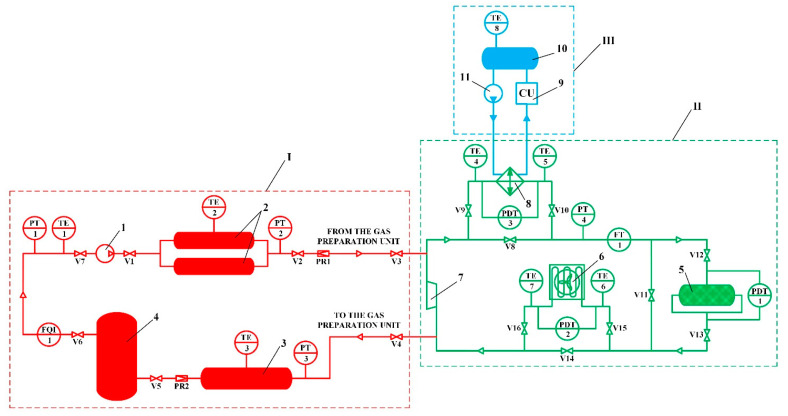
Schematic diagram of the experimental setup for study of the thermal management process during circulating charging of the ANG storage system. I—high pressure gas preparation unit; II—circulating charging unit; III—coolant preparation unit; 1—natural gas compressor; 2—high pressure tank; 3—medium pressure tank; 4—low pressure tank; 5—ANG storage system (adsorption tank); 6—“natural gas–air” heat exchanger; 7—supercharger; 8—“natural gas–ethanol” heat exchanger; 9—cooling unit; 10—ethanol tank; 11—circulation pump; PR1–2—pressure regulators; V1–16—stop valves; PT1–4—pressure sensors; PDT1–3—differential pressure sensors; TE1–8—temperature sensors; FT1—flow sensor; FQI1—gas flow meter.

A schematic diagram of the experimental setup for study of the thermal management process during circulating charging of the adsorbed natural gas storage system is presented in Figure 3.

The natural gas used in the experiments was pretreated in the high-pressure gas preparation unit I. The main element of this unit is the natural gas compressor 1, which provides the required pressure up to 25 MPa. The compressor capacity was 14 m^3^/h. The suction pressure of the compressor is monitored by a PT1 pressure sensor. After compression, the gas is accumulated in the high-pressure tank 2, which consists of two straight-through cylinders with a volume of 65 L each. This tank stores gas at high pressure between experimental studies and supplies gas during experiments. The pressure in this tank is monitored with a PT2 pressure sensor. A medium-pressure tank 3 with a volume of 300 L was designed for storing gas at intermediate pressure, as well as for ensuring safety when working at the experimental setup, since it is the recipient for gas from the entire setup. The pressure in this tank is monitored with a PT3 pressure sensor. The main function of the low-pressure tank 4 with a volume of 430 L is to smooth out pulsations of the gas flow at the suction to the compressor. The pressure in this tank is monitored with a PT1 pressure sensor. Before the experiments, the experimental setup was calibrated with the determination of the volumes of all its elements using an FQI1 gas flow meter and corresponding pressure and temperature sensors.

In this scheme, the adsorption heat released during charging of the system is removed using cooled natural gas, which flows through the adsorbent layer of the adsorption tank 5 with a volume of 51 L. This tank has one inlet and two outlets for the gas flow distribution, which makes it possible to reduce the hydraulic losses by up to 8 times with the same adsorber diameter. In fact, the flow was divided nonuniformly, which reduced the effectiveness and feasibility of this solution and made it difficult to analyze the results of the study. Gas circulation is provided by a supercharger 7. During accumulation, new portions of gas enter the circuit from the gas preparation unit I through the pressure regulator PR1 and valve V3. The charging process is carried out in two stages: the organization of isothermal charging with the heat removal into the environment (stage 1) and low-temperature charging with additional heat removal using a cooling unit III (stage 2). During isothermal charging, the heat of adsorption is removed using a “natural gas–air” heat exchanger 6: valves V2, V3, V8, V10, V12, V13, V15 and V16 are open, while valves V1, V4, V5, V6, V7, V9, V11, V14 are closed. During low-temperature charging, the heat of adsorption is removed using a “natural gas–ethanol” heat exchanger 8 to the ethanol cooled by a coolant preparation unit III: valves V2, V3, V9, V10, V12–V14 and V16 are open, while valves V1, V4, V5, V6, V7, V8, V11 and V15 are closed. The ethanol tank 10 is a “cold accumulator” to speed up the low-temperature charging process. The circulation of ethanol is provided by a circulation pump 11.

The pressure in the circuit is controlled by a pressure sensor PT4 and is provided by a pressure regulator PR1. The flow rate of the circulated gas is measured by the FT1 flow sensor and is controlled by changing the supercharger rotation speed. Hydraulic losses in the adsorber and heat exchangers are measured using differential pressure sensors PDT1, PDT2, and PDT3, respectively. Temperature measurement at the ends of heat exchangers is carried out using temperature sensors TT4–7.

The experimental setup is a closed system. The replacement of natural gas is accompanied by the regeneration of the ANG storage system, which avoids the significant accumulation of gas impurities in the adsorbent.

**Figure 4:**
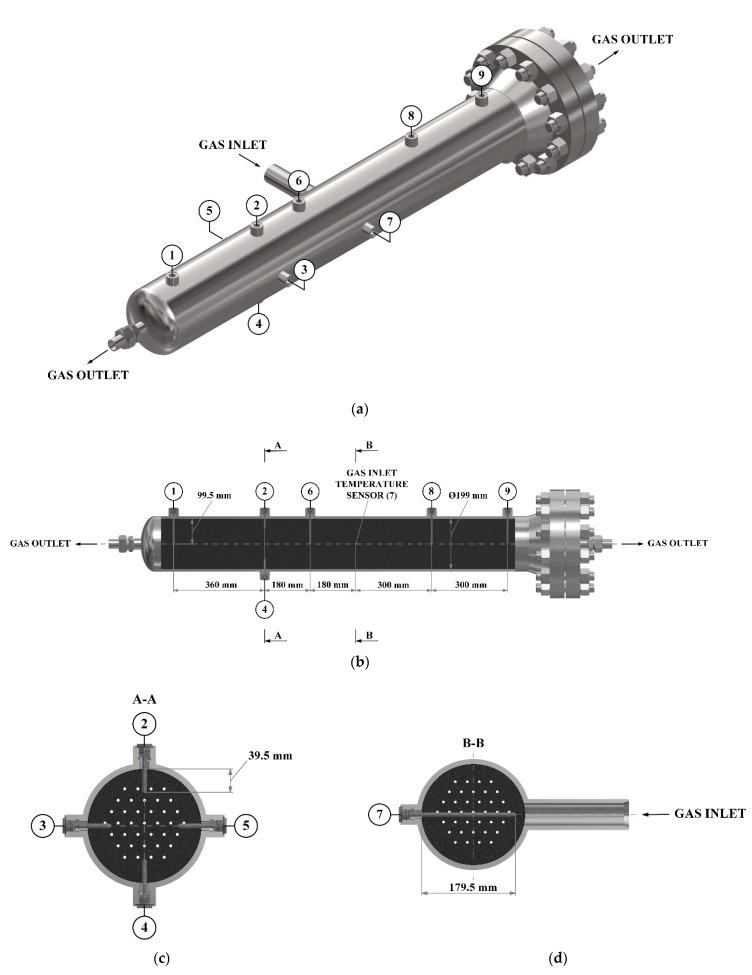
Scheme of temperature sensors (1–9) located inside the adsorption tank (**a**) and coordinates of the temperature sensors location (**b**–**d**).

To control the temperature distribution inside the heat-insulated adsorber, temperature sensor tubes were installed in it. The installation diagram of these sensors is shown in Figure 4. Sensitive elements of temperature sensors 1, 6, 8, and 9 are located along the axis of the adsorber, the sensitive element of the sensor 7 is located at the inlet to the adsorber. Temperature sensors 2–5 are located to evaluate the radial temperature gradient. The average temperature of the adsorption layer was determined under the assumption of the linearity of the axial temperature gradient in both parts of the adsorber (relative to the inlet pipe), and also under the assumption that the radial temperature gradient is approximately the same along the entire length of the adsorber.

### 2.4. Experimental Methods

A series of thirteen experiments was carried out in a two-stage circulating charging of the ANG storage system. To provide reliable initial data for subsequent research including mathematical modeling, the circulation circuit and heat exchangers were not previously cooled. Their initial temperature and the temperature of the ANG accumulator were close to the ambient temperature, i.e., reliably known. A long period of reaching equilibrium was ensured between the stages.

In eight experiments, the filling pressure and the volumetric flow rate of circulated natural gas were kept constant (with a deviation of less than 3%) and ranged from 0.5 to 3.5 MPa and from 8 to 18 m^3^/h, respectively. In five more experiments at various pressures (from 1.2 to 3.5 MPa), a constant flow mode was maintained in accordance with the Reynolds number in the inlet section of the adsorber (from 100,000 to 220,000 with a deviation of less than 3%). These experiments were carried out with the aim of a more in-depth study of heat and mass transfer processes, excluding errors due to a change in the flow mode during the cooling of natural gas and the adsorber.

To evaluate the efficiency of charging in various modes, a number of macroscopic indicators were used, which were determined for the whole adsorber and did not take into account local deviations or differences between the two parts of the adsorber separated by the inlet pipe. The main characteristic of practical importance is the storage capacity of the system. Effective charging capacity *ΔV_a_*, i.e., the excess amount of loaded gas in relation to the initial state, was empirically estimated by the “volumetric method” using the gas mass balance in the experimental setup:

(1) $$\Delta V_{a}=-\sum^{\;}\Delta V_{i}-\Delta V_{loss},$$

where $\Delta V_{i}$ is the change in the amount of gas in all elements of the experimental setup except the adsorber (m^3^ (STP)), where STP is the standard temperature (20 °C) and pressure (101,325 Pa); and $\Delta V_{loss}$, (m^3^ (STP)), is the gas leaks determined by the rate of decrease in the amount of gas in the experimental setup under the conditions of the closest achievement of the equilibrium of heat and mass transfer in the adsorber, i.e., when the adsorber cooling has slowed down to a minimum level. 

The theoretical charging capacity was determined using the current pressure and temperature at a given point in time and data from previous experimental studies of the adsorption properties of AU-1 [24,29,37,38]. The average temperature of the adsorbent was determined by temperature sensors located inside the adsorber.

Charging speed can be estimated using the effective circulating charging time *τ_e_*, which is counted from the supercharger (circulation) start (differs in experiments) and determined in the experiment by the acceptable final temperature difference Δ*T_e_* between the incoming gas flow and the average temperature of the adsorbent. The value of Δ*T_e_* determines the direct loss in the storage capacity of circulating charging. A certain effective charging time corresponds to an acceptable loss in storage capacity. For example, the final temperature difference of 5 K corresponds to the theoretical [24] loss of effective charging capacity in this adsorber from 3.5% at 3.5 MPa to 10% at 0.5 MPa, or in absolute values, about 0.12–0.20 m^3^ (STP).

The efficiency of the heat transfer in the adsorber can be estimated by analogy with heat exchangers using the heat exchange efficiency coefficient *K_he_*, which is determined by the ratio between the actual heat flux and the maximum heat flux corresponding to an infinitely high heat transfer coefficient or an infinitely large heat exchange surface. The actual heat flux was determined from the theoretical dependences presented in [24,30] according to the rate of decrease in the measured temperatures of the adsorbent layer. Coefficient *K_he_* characterizes the balance between the charging mode and the surface area (structure) of the adsorbent monoliths.

(2) $$K_{he}=\frac{Q}{Q_{max}}=\frac{h_{g2}-h_{g1}}{{h_{g}|}_{T=T_{a2}}-h_{g1}}=\frac{\frac{\partial H_{a}}{\partial T_{a}}\cdot \frac{dT_{a}}{d\tau }}{G\cdot \left({h_{g}|}_{T=T_{a2}}-h_{g1}\right)},$$

where Q is the actual heat flux, (W); $Q_{max}$, (W), is the maximum possible heat flux determined by the mass gas flow rate G, the specific enthalpy of the gas at the inlet $h_{g1}$ and the specific enthalpy of the gas corresponding to the temperature of the adsorbent at the outlet of the adsorber ${h_{g}|}_{T=T_{a2}}$; $h_{g2}$ is the specific enthalpy of gas at the outlet of the adsorber; τ is the time, (s); $T_{a}$ is the average temperature of the adsorption layer, (K); and $H_{a}$ is the total enthalpy of the adsorber, consisting of the enthalpies of the adsorbent (with a binder), gas component, and metal parts of the adsorber, (J). 

Since the two parts of the adsorber, separated by the inlet pipe, differed from each other, the heat exchange efficiency coefficient was determined from the sums of heat fluxes in each part of the adsorber:

(3) $$K_{he}=\frac{Q_{L}+Q_{R}}{Q_{maxL}+Q_{maxR}},$$

where the indices “L” and “R” correspond to the “left” and “right” sides of the adsorber.

Evaluation of the heat transfer coefficient in this experiment was not a main target task and, in fact, was a by-product of the study of integral processes of charging a full-size adsorber. The value of the heat transfer coefficient $K_{ht}$ was estimated by indirect methods: the heat flux removed from the adsorbent was determined from the adsorbent temperature change (as in the case of determining $K_{he}$) and the logarithmic mean temperature difference was determined from the experimentally determined temperatures of the adsorbent layer and the inlet gas flow with outlet gas temperature calculated from the heat balance and with corrections using the adsorbent temperature gradient along the adsorber axis. In order to exclude possible errors associated with the radial temperature gradient and heat transfer in the gap between the monoliths and the walls of the adsorber, in which the metal parts of the adsorber and the environment also participate, only the adsorber regions close to the axis were considered.

The value of the heat transfer coefficient was estimated by the following equation, (W/(m^2^∙K)):

(4) $$K_{ht}=\frac{q_{V}}{f_{V}\cdot \Delta T_{log}}\approx \frac{\frac{\partial \left(h_{aV}+h_{g}\cdot \varepsilon \cdot \rho_{g}\right)}{\partial T_{a}}\cdot \frac{dT_{a.ax}}{d\tau }}{f_{V}\cdot \frac{\left(T_{a.ax1}-T_{g1}\right)-\left(T_{a.ax2}-T_{g2}\right)}{\ln\left(\frac{T_{a.ax1}-T_{g1}}{T_{a.ax2}-T_{g2}}\right)}},$$

where $q_{V}$ is the heat flux reduced to the volume of the adsorbent, (W/m^3^); $h_{aV}$ is the enthalpy of the adsorption layer, reduced to the volume of the adsorbent, (J/m^3^); $h_{g}$ is the specific enthalpy of the gas component in monoliths, (J/kg); ε is the porosity, fraction of free space for the gas component; $\rho_{g}$ is the gas density, (kg/m^3^); $T_{a.ax}$ is the average temperature of the adsorption layer in the vicinity of the adsorber axis, (K); $\Delta T_{log}$ is the logarithmic mean temperature difference between the gas flow and the adsorption layer, (K); $T_{g1}$ and $T_{g2}$ are the gas temperatures at the inlet and outlet of the adsorber, respectively, (K); $T_{a.ax1}$ and $T_{a.ax2}$ are the temperatures of the adsorbent along the axis of the adsorber at the inlet and outlet from it, (K); and $f_{V}$ is the specific heat transfer surface in the vicinity of the adsorber axis (excluding the outer cylindrical surface of the monoliths), reduced to the volume of the adsorbent, (m^2^/m^3^).

The Reynolds number of the flow can be reduced both to the total section of the adsorber *Re_f_*, and to the “narrow” section (in channels), taking into account the space occupied by the adsorbent *Re_c_* ≈ 0.48∙*Re_f_* (the hydraulic diameter was equal to the channel diameter: 4.5 mm). Due to the complex nature of heat transfer, the correct choice of the Reynolds number is difficult: *Re_f_* is more reasonable for heat transfer with the edge surface, and *Re_c_* corresponds to heat transfer in the channels.

### 2.5. Mathematical Model

Due to the small number of sensors installed in the experimental setup, as well as due to the complex nonuniform gas flow through monoliths, it is impossible to determine the heat transfer coefficients with high accuracy. However, it is possible to determine the general patterns of their change, which can be used in practice, particularly in the development of a mathematical model of the cooled circulating charging process.

It was proposed to introduce a simple mathematical model, at the same time correlated with the experiments performed. This mathematical model makes it possible to calculate real charging processes in such systems, though only with the use of monoliths of the same shape and size as in this study. A more general solution is possible in the future when studying monoliths of other shapes and sizes.

The mathematical model is one-dimensional. The adsorption layer consisting of a number of monoliths is reduced to a layer uniform over charging, divided into *n* cells. The heat transfer area is also considered uniform in the volume of the layer. Heat transfer processes inside the channels and the outer surfaces are not separated from each other. The charging process is isobaric, which greatly simplifies the solution. We neglected the processes of longitudinal (along the axis) diffusion and thermal conductivity. We also neglected the rate of mass transfer, i.e., we considered the adsorption process to be instantaneous. Also, for simplicity, only the central (along the axis of the adsorber) adsorbent layer was considered, which made it possible to neglect the heat exchange with the environment, as well as the influence of the metal parts of the adsorber. All parameters of the adsorption layer are uniform inside each cell. The general calculation of the model is reduced to a sequential calculation for each cell. This approach makes it possible, with a small loss of accuracy, to simulate the charging process of a long adsorber by simulating the *N* number of monoliths, which can be represented as a cell of this model. Since monoliths are often installed with some gaps between them, neglecting the influence of neighboring monoliths fits well with the simplifications of this model. For simpler calculations, the entire adsorber can be considered as a cell, i.e., the adsorber can be calculated by analogy with simple heat exchangers. However, in this study, to improve accuracy, the cell size was an order of magnitude smaller than monoliths.

The temperature of the gas leaving the cell $T_{gc2}$ is determined by the equation, (K):

(5) $$T_{gc2}=T_{ac}-\left(T_{ac}-T_{gc1}\right)\cdot e^{-K_{ht}\cdot f_{V}\cdot \frac{\Delta x}{\upsilon_{gc1}\cdot \rho_{gc1}\cdot c_{gc1}}},$$

where $T_{ac}$ is the current temperature of the adsorbent in the cell (to improve accuracy, it is better to use the average between the start and final temperatures at a given step, which leads to the method of successive approximations), (K); $T_{gc1}$ is the temperature of gas entering the cell, (K); Δx is the longitudinal cell size, (m); $\upsilon_{gc1}$ is the velocity of gas entering the cell, reduced to the cross-sectional area of the cell, (m/s); $\rho_{gc1}$ is the density of gas entering the cell, (kg/m^3^); and $c_{gc1}$ is the specific isobaric heat capacity of gas entering the cell, (J/(kg∙K)).

This equation is simplified due to neglect of the fraction of the gas flow adsorbed in the cell relative to the circulation flow. In fact, in the entire adsorber, only a few percent or fractions of a percent of the circulating flow are adsorbed, and in individual cells this fraction is negligible.

Specific heat flux (reduced to the cell section area) in the cell is determined by the temperatures of the entering and leaving gas flows, (W/m^2^):

(6) $$q_{c}=\upsilon_{gc1}\cdot \rho_{gc1}\cdot c_{gc1}\cdot \left(T_{gc2}-T_{gc1}\right),$$

Note that the cell section area does not play a role in this model, and integral quantities like heat flux or enthalpy are reduced either to the cell section area or to its volume.

The change in the temperature of the adsorbent in the cell with time is determined in accordance with the heat flux:

(7) $$\frac{dT_{ac}}{d\tau }=-\frac{q_{c}}{\Delta x\cdot \frac{\partial h_{aV}}{\partial T_{ac}}},$$

A change in the temperature of the adsorbent corresponds to a change in the amount of adsorbed gas and the specific enthalpy of the adsorbent, in accordance with the procedure described in [24,30]. Despite the small fraction of the circulating flow that is absorbed in the cell, in subsequent cells it is advisable to consider the change in the gas flow rate:

(8) $$-\Delta T_{ac}\cdot \frac{\delta m_{aV}}{\delta T_{ac}}\cdot \Delta x=\left(\upsilon_{gc2}\cdot \rho_{gc2}-\upsilon_{gc1}\cdot \rho_{gc1}\right)\cdot \Delta \tau ,$$

where $m_{aV}$ is the specific capacity of the adsorption layer, reduced to the volume of the adsorbent, (kg/m^3^); $\upsilon_{gc2}$ is the velocity of the leaving gas flow, (m/s); $\rho_{gc2}$ is the density of the leaving gas flow, (kg/m^3^); and Δτ is the simulation time step, (s).

In this work, the method of successive approximations was used to improve the accuracy of the solution; however, when choosing small steps Δx and Δτ, most of the values from iteration to iteration changed very weakly, which made it possible to perform calculations with an acceptable accuracy in one step.

The heat transfer coefficient $K_{ht}$ required for the model was estimated in the first approximation from the experimental data (but with significant errors) and was further corrected in accordance with the model’s calculations.

## 3. Results and Discussion

Table 1 and Table 2 provide brief information on the main investigated charging modes with constant pressure and volumetric gas flow or gas flow mode, as well as a number of results obtained. The results of the study showed the correlation between the charging mode and the indicators of the charging efficiency. Experiment 1 achieved the smallest effective charging time, which ranged from 12 to 20 min, depending on the charging stage. In experiment 2, a 1.5-fold decrease in gas flow rate led to an increase in the charging time by almost 1.5 times. Experiment 3 also showed a similar charging time due to a similar flow mode: Reynolds numbers in these two modes were close to each other. However, there were also differences; the isothermal charging in experiment 3 was noticeably faster (13 min versus 17 min in experiment 2), which was due to a smaller amount of accumulated gas and, accordingly, a lower release of adsorption heat. The low-temperature charging in experiment 3 was slightly slower, which was possibly due to experimental errors: mainly, the irreproducibility of the dynamics of the supplied gas temperature in different experiments, as well as an insufficiently detailed measurement of the thermal field inside the adsorber. In general, it can be noted that with a decrease in gas flow rate, the effective charging time significantly increases. A decrease in the charging pressure has a negative effect on the duration of low-temperature charging and has little effect on the duration of isothermal charging due to a decrease in the adsorption heat generated in the initial period of charging. At the same time, from the standpoint of the effective storage capacity, the low-temperature charging of the adsorber based on AU-1 is more expedient, specifically at low pressures: at 0.5 MPa, the increase in the amount of gas with cooling was 116% and, at 3.5 MPa, it was only 37–40%. However, the low speed of charging at low pressure significantly limits the areas of practical application of low-temperature charging.

The highest achieved effective storage capacity in the experiment was 4.88–5.12 m^3^ (STP) with isothermal charging and 6.71–7.16 m^3^ (STP) with low-temperature charging. The differences are probably due to the existing measurement errors (the amount of gas was determined by subtracting the gas volumes in all the elements of the setup, i.e., the errors from each element were summed up), unaccounted for unstable leaks, and the nonuniform accumulation of impurities from natural gas. In specific terms, the effective capacity was 96–100 m^3^ (STP)/m^3^ for isothermal charging and 132–140 m^3^ (STP)/m^3^ for low-temperature charging. This value does not include the initial amount of gas (about 20 m^3^ (STP)/m^3^), part of which can be extracted during desorption with heating the adsorbent. In addition, the adsorbent filled approximately 80% of the internal volume of the adsorber, so that when the charging technique is refined, a specific effective storage capacity of 175 m^3^ (STP)/m^3^ can be reached in the limit. The results in Table 2 correspond to those in Table 1 and confirm the above conclusions. However, in practice, it is difficult to implement charging with a constant flow mode (Reynolds number). Experiments 10 and 11 were very close to each other in terms of low-temperature charging indicators, which was expected given their identical flow modes. Differences in isothermal charging are explained by different pressures.

The value of *K_he_* in all the experiments was rather high (from 0.62 to 0.83), which indicates an acceptable surface area of monoliths for these charging modes. This is a somewhat unexpected result, since it was assumed that the small number of channels in the monoliths might not be enough for efficient heat transfer. Experiment 5 had the highest heat exchange efficiency coefficient. This is likely due to the combination of both high pressure, which provides intense heat exchange, and low gas flow rate, which ensures sufficient gas heating.

**Figure 5:**
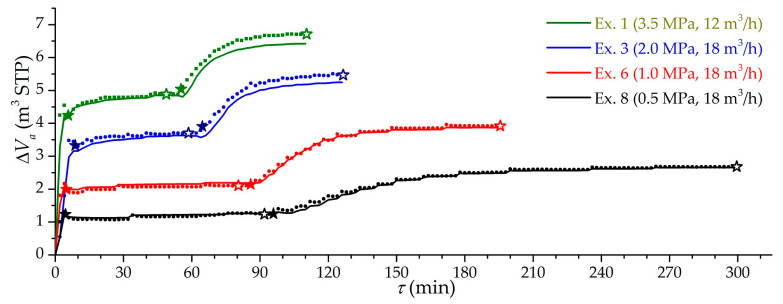
Time dependence of the effective charging capacity at different modes (the number of the experiment is indicated) at isothermal (stage 1) and low-temperature charging (stage 2). Lines are theoretical values, dots are experimental values, solid “stars” are the beginning of circulating charging, and open “stars” are the end of circulating charging.

Figure 5 shows the dependencies of the effective charging capacity for four modes of charging at pressures from 0.5 to 3.5 MPa. Symbol “stars” show the moments of the supercharger turning on and off. The experimental and theoretical values of the effective charging capacity are in good accordance. The existing differences are due to the unaccounted component of leaks, the accumulation of impurities from natural gas, and errors of the volumetric method. Experimental capacity fluctuations are explained by dynamic errors in the used volumetric method. A much slower charging in low-temperature mode was mainly due to the cooling down of both the stand itself and the metal parts of the adsorber.

**Figure 6:**
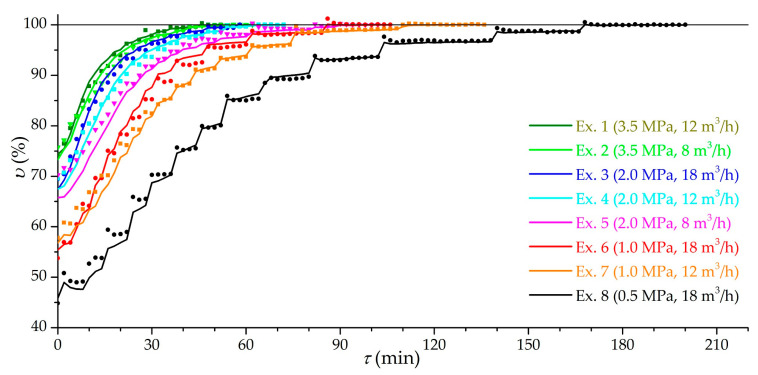
Time dependence of the relative effective charging capacity at different modes (the number of the experiment is indicated) at low-temperature charging (stage 2). Lines are theoretical values and dots are experimental values.

Figure 6 shows the dynamic dependencies of the relative capacity *υ* of the low-temperature charging for the main group of experiments (from 1 to 8), while the achieved effective charging capacity in the experiment is taken as 100%. In relative coordinates, excluding leakage and the accumulation of impurities, the correspondence between the theoretical amount of gas and the experimental storage capacity is more obvious. The stepped shape of the curves is due to the supply of gas in portions. This figure clearly shows the difference in the duration of low-temperature charging in different modes. Charging 80% of the maximum can take from 4 min at 3.5 MPa, in experiments in which low-temperature charging gives a small increase in the amount of gas, up to 52 min at 0.5 MPa, and 90%, respectively, from 13 min at 3.5 MPa to 80 min at 0.5 MPa. At a pressure of 1.0 MPa (experiments 6 and 7), a good balance can be observed between the duration of the low-temperature charging and the increase in the amount of gas (up to 1.9 times). This pressure is interesting for practical application in the case of direct gas supply from the gas distribution network or with preliminary compression in a one- or two-stage compressor.

**Figure 7:**
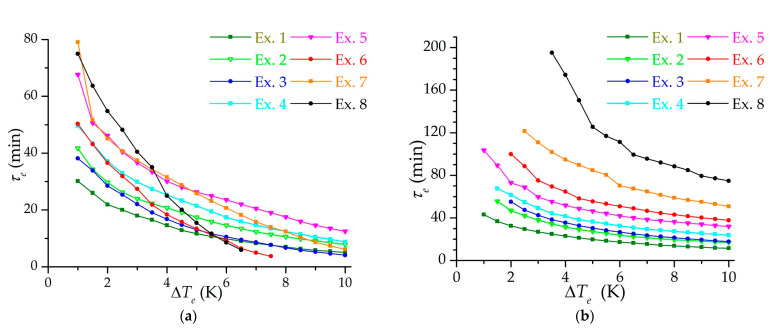
Dependence of the effective charging time on the final temperature difference between the inlet gas and the adsorbent for isothermal (**a**) and low-temperature charging (**b**) at different modes (the number of the experiment is indicated).

Figure 7 shows the dependencies of the effective charging time on the final temperature difference between the inlet gas and the adsorbent (in a range from 1 to 10 K). Experiments 2 and 3, as well as experiments 5 and 6, showed similar results in the low-temperature charging mode (Figure 7a), since they have practically the same flow modes (according to Reynolds numbers): lower pressures and density correspond to higher flow rates. However, in experiments with lower pressure, less heat of adsorption is released, which is noticeable in the isothermal charging mode (Figure 7b). It can be seen from the figure that it is possible to achieve a five-minute charging in the isothermal mode only with undercooling from 7 K and higher. In the low-temperature mode under these conditions, a five-minute charging is practically unattainable with a moderate choice of the final temperature difference. This is due to the cooling of the metal parts of the adsorber and the experimental setup itself. Thus, these dependences are not a characteristic of the adsorber or adsorbent, but of the experimental setup as a whole system: the duration of charging and the cooling rate largely depend on the processes taking place outside the adsorber.

**Figure 8:**
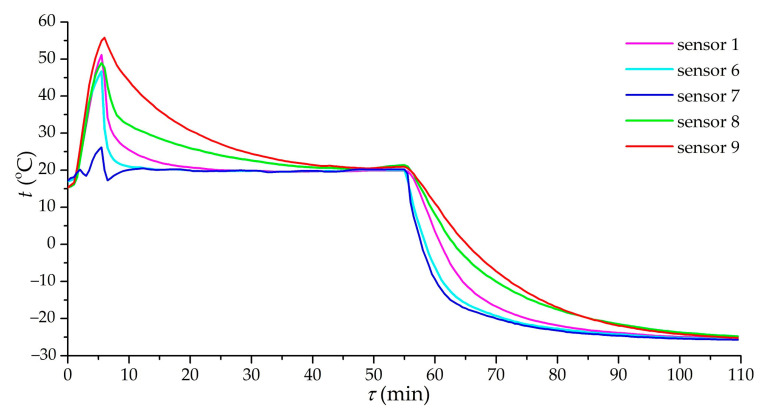
Dependence of the temperature of the sensors (1, 6–9) on time during charging. Charging mode: pressure of 3.5 MPa and volumetric gas flow rate of 12 m^3^/h.

Figure 8 shows the dependence of the temperature at various points of the adsorber on time in the case of charging at a pressure of 3.5 MPa and a volumetric gas flow rate of 12 m^3^/h (Experiment 1). There is a clear heterogeneity in the cooling of one half of the adsorber compared to the other: the adsorbent in the area of sensors 1 and 6 is cooled much faster than in the area of sensors 8 and 9. Ensuring the uniformity of gas flow distribution seems to be one of the most difficult technical problems in real systems: dust of the adsorbent, clogging filters, and so on can significantly distort the distribution of flows. It was found that the approximate ratio of gas flow rates was 2.5:1, and it completely changed the nature of the adsorber cooling and, probably, increased the duration of charging.

**Figure 9:**
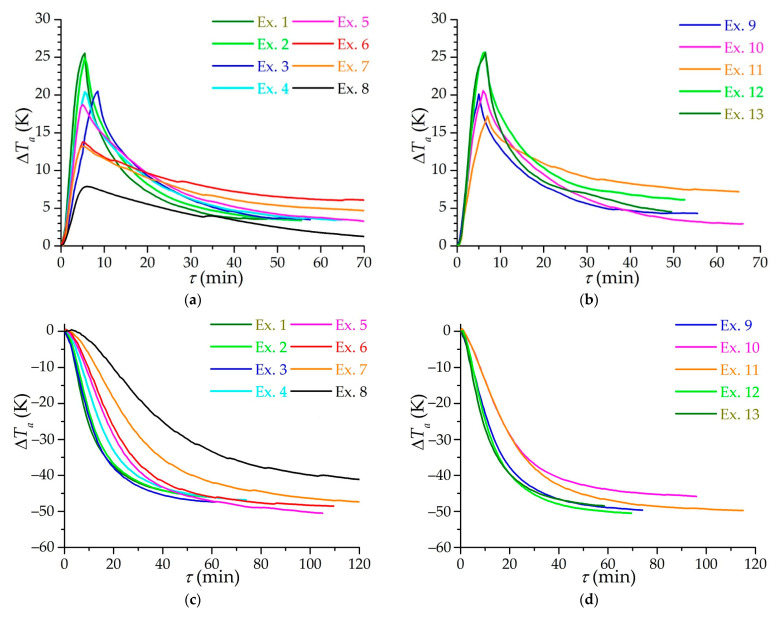
Time dependence of the adsorbent average temperature change during isothermal (**a**,**b**) and low temperature (**c**,**d**) circulating charging. Subfigures (**a**,**c**) refer to the main group of experiments (from 1 to 8), and subfigures (**b**,**d**) to additional experiments (from 9 to 13) with a constant flow mode.

Figure 9 shows the dependencies of the change in the average temperature of the adsorbent layer on time for various modes of charging. Due to different initial temperatures and temperatures of the ambient air, graphs are presented specifically for the change in the average temperature (the initial temperatures of the adsorbent are taken as 0 before isothermal and low-temperature charging). It should be noted that the average temperature of the adsorbent is determined approximately, being in fact an “apparent” temperature: the installed temperature sensors are influenced by the circulating flows of a gas having a temperature different from the temperature of the adsorbent; in addition, the number of sensors is not enough to accurately determine the average temperature in such a large volume of the adsorption layer (51 L), so the average temperature is determined from the assumptions about the linearity of the axial temperature gradient and a constant radial gradient along the entire adsorber. Peak heating during isothermal charging (Figure 9a,b) is practically the same in all experiments with equal pressure: for example, in experiments 1, 2, 12, and 13. Small differences arise at different rates of gas supply to the system as in the case of experiments 3–5. It can be assumed that the peak heating during isothermal charging is underestimated relative to the actual heating of the adsorbent due to the inertia of the temperature sensors.

Figure 9c,d shows similar dependencies for low-temperature charging. Here the closeness of the results of experiments 2 and 3 can be noted, as well as those of experiments 5 and 6, which are pairwise close to each other in the flow mode (Reynolds number). Figure 9d shows the dependencies for experiments with maintaining the flow mode at the inlet: experiments with the same Reynolds number at the inlet 10 and 11, as well as 9 and 12, also show very similar results. The discrepancy with time between experiments 10 and 11 is explained by different initial temperatures of the process—in experiment 10 the initial temperature is lower, and the final temperature is almost the same, i.e., the process tends to change the temperature differently compared to experiment 11. Experiments 1 and 13 are knocked out of the general patterns and turned out to be close, respectively, to experiments 2 and 3 and 9 and 12. In this case, the probable influence of the experimental setup is evident: the refrigeration capacity (the flow of the coolant in the “methane–ethanol” heat exchanger) is not enough to ensure faster charging, although the heat exchange process itself in the adsorber is more efficient, which can be detected by estimates of the heat transfer coefficient.

The study of heat exchange processes at the micro level was not initially included in the experiment. This is noticeable by the small number of temperature sensors installed in the adsorber and a number of simplifications used, such as a linear axial temperature gradient. This resulted in rather large errors in the experimental heat transfer coefficients. However, the experiment made it possible to evaluate a number of patterns in the change in the heat transfer coefficients, which in turn made it possible to implement a simple one-dimensional mathematical model of the adsorber charging. The process of heat transfer in the adsorbent layer in this case is rather complicated, since the final result is influenced by heat transfer on the surface of the cylindrical channels; heat transfer along the end surfaces of monoliths; thermal conductivity of the adsorbent layer; mass transfer providing gas movement through the adsorbent layer; heat release in the adsorbent itself, taking into account possible accumulations of impurities; heat leakages from the environment; heat capacity of the adsorber tank itself, especially from the flange side, and so on. The nonuniform distribution of the gas flow with its possible slip through the radial gaps between the monoliths and the adsorber walls has a negative impact on the accuracy of prediction and the determination of experimental heat transfer coefficients. Also, there are temperature measurement errors, including inertia of the temperature sensors, and simplifications in the experimental and data processing methodology, such as a linear axial temperature gradient. Considering all of the above, further results should be considered approximate and estimated, although they can be used in practice with appropriate safety factors when calculating the adsorber and the charging process with a similar working substance and a similar adsorbent (with the same adsorption properties and monolith geometry).

Analysis of the approximate experimental heat transfer coefficients $K_{ht}$ showed the direct influence of the flow mode (in the form of the Reynolds number) and the rate of relative change in the gas flow temperature, which is an external (to the adsorbent) criterion of the dynamic process. The second component characterizes the rate of restructuring of the thermal field inside the adsorbent. Thus, during periods of rapid change in the temperature of the gas flow, the walls of the adsorbent in contact with it do not have time to change the temperature so quickly, which leads to a noticeable temperature difference between the surface of the adsorbent and the gas that provides intense heat exchange associated with the rearrangement of the thermal field in the adsorbent. If the temperature of the gas flow changes weakly, then the thermal field inside the adsorbent is rearranged so that the surface temperature is slightly different from the temperature of the gas flow, and the main limiting factor for heat transfer is the thermal conductivity of the adsorbent, which significantly reduces the intensity of heat transfer. This approach is very simplified: for example, it does not take into account the “history” of changes in the thermal field in the adsorbent, and therefore it can give strange results with fluctuating changes in the temperature of the supplied gas. Also, in this experiment, it was not possible to establish the effect of the working substance properties (in the form of the Prandtl number, etc.) and the adsorbent (thermal conductivity and diffusivity, etc.) on heat transfer due to the fact that only one adsorbent and one working substance were used in the experiment. Problems also occurred when trying to take into account the effect of mass transfer on heat transfer due to their association: intense heat transfer leads to rapid cooling of the adsorbent, which in turn is accompanied by intense mass transfer. It is possible to separate the effect of mass transfer on heat transfer only in subsequent experiments using adsorbents with other adsorption properties or porous non-adsorbing materials.

Analysis of the estimated experimental heat transfer coefficients $K_{ht}$ led to the following empirical expression, which is in good agreement with both the experiment and the mathematical model, (W/(m^2^∙K)):

(9) $$K_{ht\left(model\right)}=0.00824\cdot Re_{f}^{0.78}\cdot \left(\frac{1+{\left(\theta /\theta_{min}\right)}^{0.69}}{2}\right),\;\theta_{min}=-1.1\cdot 10^{-4}\;s^{-1},\;3200<Re_{f}<43000,\;\theta <0,\;\left|\theta_{min}\right|<\left|\theta \right|<0.02\;s^{-1},$$

where θ is the rate of relative change in gas flow temperature $T_{g}$, defined as (s^−1^):

(10) $$\theta =\frac{dT_{g}}{d\tau }/\Delta T_{log},$$

The limits of applicability of Equation (9) are determined by the conditions achieved in the experiment. If Equation (9) is given a physical meaning, then $\theta_{min}$ is the limit for the appearance of the dynamic component of the heat transfer coefficient. For smaller |θ|, the last term of Equation (9) can be neglected (the factor is 1). In the experiment, it was impossible to maintain $\left|\theta \right|<\left|\theta_{min}\right|$; therefore, the empirical Equation (9) corresponds to the experiment only in the region $\left|\theta \right|>\left|\theta_{min}\right|$ (and corresponds even more reliably for $\left|\theta \right|>5\cdot \left|\theta_{min}\right|$). Equation (9) is not intended for cases where the gas temperature increases, because only the analysis of cooling charging results was carried out. High values of |θ|, above the indicated limits of applicability, showed themselves in the initial periods of cooling, at very small values of $\Delta T_{log}$; however, these time periods were excluded from the analysis due to the significant dynamic errors of the experiment. Among the disadvantages of using θ, it can be noted that this criterion is not dimensionless, and attempts to bring it to a dimensionless form were unsuccessful (there was probably not enough data and other experiments).

Equation (9) can only be used in the case of a similar adsorbent and working substance. The advantage of Equation (9) is its simplicity. It is also worth noting the value of the degree at the Reynolds number of 0.78, which is very close to the typical degree of 0.8 in heat transfer processes in turbulent flow. However, this also reveals the drawback of Equation (9), which is that it does not consider different types of flow, since most of the experiments fell into the region of turbulent and transient flows, and very little into the region of possible laminar flow in channels (at the same time, the geometry of the adsorbent and high surface roughness maximizes the turbulence of the flow).

**Figure 10:**
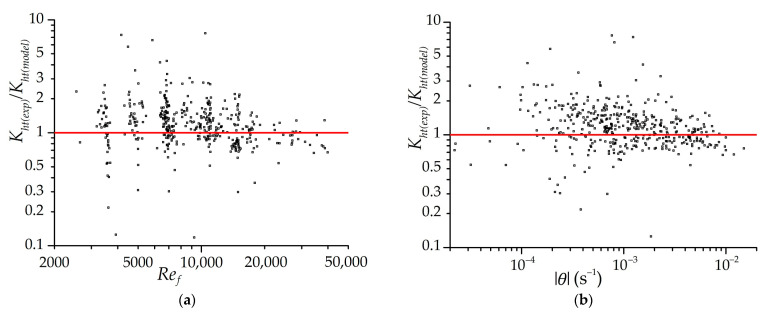
The distributions of the ratio of the estimated experimental heat transfer coefficients to the coefficients determined by Equation (9) depending on the Reynolds number (**a**) and the relative change in the gas temperature θ (**b**). Dots are separate measurements. The line is an exact match to the empirical Equation (9).

Figure 10 shows the distributions of the ratio of the estimated experimental heat transfer coefficients to the coefficients determined by Equation (9). The dependencies on the Reynolds number and the rate of the relative change in the gas temperature θ in the experiment are presented. The experimental heat transfer coefficients are determined with a minimum time step of 120 s. Figure 10 shows the most reliable area of distribution of heat transfer coefficients, for example, when the temperature difference between the adsorbent and gas is not less than 3 K (to reduce the influence of measurement errors). Experimental heat transfer coefficients are determined for both isothermal and low-temperature charging, with the exception of the initial periods of processes when dynamic errors are large (2 min for isothermal charging and 1 min for low-temperature charging). The “noises” on the graphs caused by the errors of the experiment and the method of processing the experimental data are very large, but the dependencies themselves fit well into the regularity indicated in Equation (9). Most of the dots are within the range from $0.5K_{ht\left(model\right)}$ to $2K_{ht\left(model\right)}$. A slight displacement of the center of distribution of points relative to 1 is explained by the fact that the coefficients in Equation (9) are corrected according to the results of mathematical modeling, considering that the experimental values contain systematic errors in data processing.

**Figure 11:**
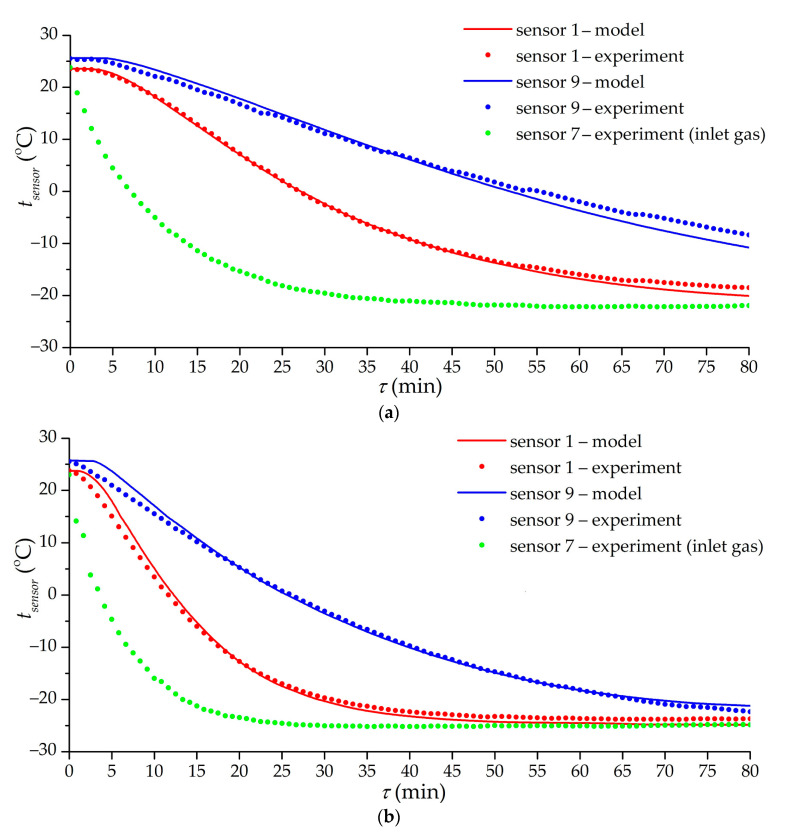
Time dependence of the temperature of sensors 1, 7, and 9 according to the results of experiments 8 (**a**) and 11 (**b**) and mathematical modeling. Lines are the results of mathematical modeling. Dots are experimental values.

Figure 11 shows the results of the mathematical modeling of low-temperature charging in modes corresponding to experiments 8 (with a constant volumetric flow rate) and 11 (with a constant flow mode at the inlet). The mathematical model included the heat transfer coefficients according to Equation (9). To compare the model and experiment, we used the readings of temperature sensors 1 and 9 in the adsorber, which are located farthest from the inlet section. This makes it possible to reduce the influence of the blurred gas supply zone in a real adsorber using the results of modeling an idealized adsorber. The simulation results adequately describe the experimental curves. At the initial moment, there is a lag in the temperature change in the model relative to the experiment, in spite of the fact that the model excludes inertia in the temperature determination. This is probably due to cold gas slip bypassing the adsorbent in a real experiment, while the model uses a more idealized heat transfer, during which the gas heats up almost to the temperature of the adsorbent and for some period cannot cool the adsorbent in the far (from the inlet) part of the adsorber. The temperature discrepancy between the model and the experiment at the end of the cooling process (as in experiment 8l; Figure 11a) is probably explained, in addition to the errors of the experiment itself, by the neglect of heat leakages from the environment in the model. This assumption does not give significant errors in most experiments (in experiment 11 it shows itself very weakly), but in experiment 8, where the cooling rate is low, the effect of heat leakage on the final temperature is significant.

**Figure 12:**
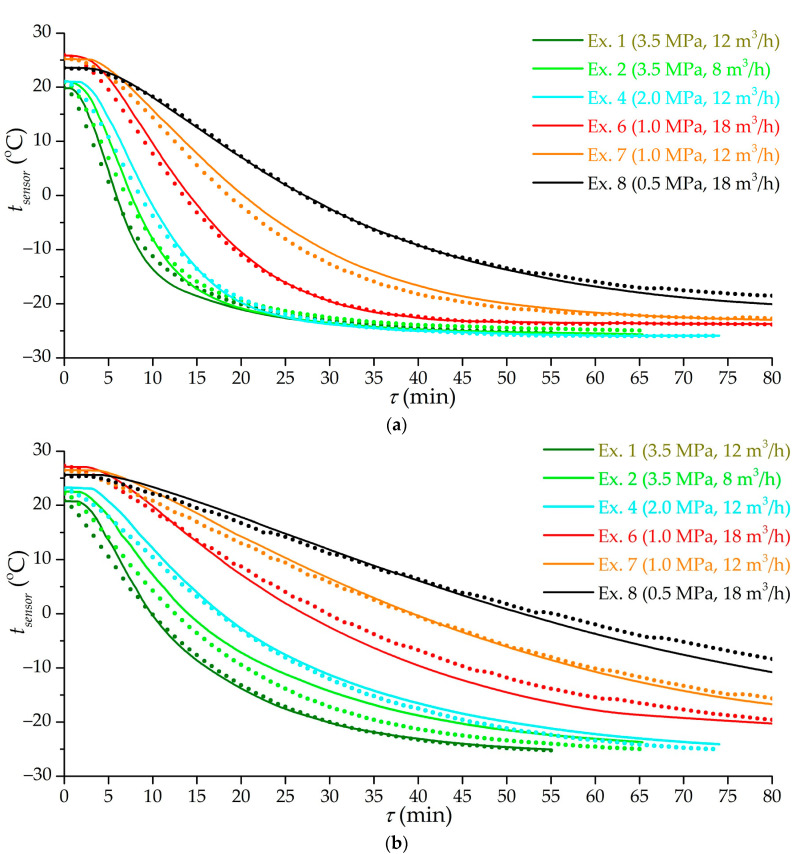
Time dependence of the temperature of sensors 1 (**a**) and 9 (**b**) according to the results of a number of experiments (the number is indicated in the figure) and mathematical modeling. Lines are the results of mathematical modeling. Dots of the same color are experimental values.

Figure 12 shows the results of mathematical modeling of the low-temperature charging process in various modes corresponding to a number of experiments. The model adequately takes into account the flow modes and the cooling rate of the gas supplied to the adsorber. The model does not take into account all the factors affecting heat transfer; therefore, further work is possible to improve the results. At the moment, the error of mathematical modeling relative to the experimental results can be estimated at 3.6 K with a confidence level of 95%, which is quite enough for the practical application of the results obtained. 

## 4. Conclusions

Studies of the process of circulating natural gas charging were carried out on a full-scale adsorber with a volume of 51 L equipped monolithic adsorbent in two modes: isothermal and low-temperature charging. The studies were carried out by providing a constant pressure (from 0.5 to 3.5 MPa) and a constant volumetric gas flow rate (from 8 to 18 m^3^/h) or a constant flow mode (Reynolds number from 100,000 to 220,000 at the inlet to the adsorber). The results of the study showed the correlation between the charging mode and the charging efficiency indicators. The fastest low-temperature charging was achieved at a pressure of 3.5 MPa and a gas flow rate of 12 m^3^/h. The highest achieved specific effective (excess in relation to the initial amount) storage capacity in the experiment was 100 m^3^ (STP)/m^3^ for isothermal charging and 140 m^3^ (STP)/m^3^ for low-temperature charging, and up to 175 m^3^ (STP)/m^3^, when reduced to the volume of the adsorbent. At a pressure of 0.5 MPa, the low-temperature charging provided a significant increase in the effective capacity of gas by 116%, but it took a very long time (80 min to reach 90% of the maximum storage capacity). The most balanced heat exchange process was observed in the charging mode at a pressure of 2.0 MPa and a gas flow rate of 8 m^3^/h: the efficiency of using the refrigerating potential of the gas flow reached 83%. In general, the heat exchange was quite efficient for the used charging modes, which indicates a sufficient heat exchange area of the monoliths. The correlation between the heat transfer coefficient and the gas flow mode for circulating charging was determined. A one-dimensional mathematical model of the adsorber charging process was developed using the experimentally estimated heat transfer coefficients between the adsorbent and the gas. The simulation results of the dynamic temperature of the sensors inside the adsorber are in adequate accordance with the experimental results (error less than 3.6 K with a confidence level of 95%).

## Figures and Tables

**Table 1:** Modes and results of the experiments with constant pressure and volumetric gas flow rate.

Parameter	Experiment							
	1	2	3	4	5	6	7	8
Pressure, MPa	3.5	3.5	2.0	2.0	2.0	1.0	1.0	0.5
Gas flow rate, m^3^/h	12	8	18	12	8	18	12	18
Initial pressure, MPa	0.13	0.13	0.12	0.13	0.14	0.14	0.13	0.14
Initial temperature, °C	16.6	18.2	19.8	18.5	21.9	20.5	21.4	23.7
Minimal temperature of adsorbent, °C	−25.2	−24.5	−22.7	−24.6	−25.4	−22.0	−22.0	−19.2
Δ*V_a_*, m^3^ STP:								
isothermal charging	4.88	5.12	3.70	3.76	3.76	2.10	2.60	1.24
full charging	6.71	7.16	5.47	5.58	5.66	3.92	4.44	2.68
*τ_e_*, min with *ΔT_e_* = 5 K:								
isothermal charging	11.6	17.4	13.0	21.4	26.3	13.4	25.8	15.5
low-temperature charging	19.7	27.2	30.4	36.5	46.4	55.5	84.8	125.4
*K_he_* with *ΔT_e_* = 5 K	0.77	0.78	0.70	0.76	0.83	0.69	0.69	0.62

**Table 2:** Modes and results of the experiments with constant pressure and gas flow mode (Reynolds number).

Parameter	Experiment				
	9	10	11	12	13
Pressure, MPa	2.0	2.0	1.2	3.5	3.5
Reynolds number at adsorber inlet	160,000	100,000	100,000	160,000	220,000
Gas flow rate, m^3^/h	13.0–20.1	7.8–11.8	13.7–19.9	7.2–11.2	10.0–15.1
Initial pressure, MPa	0.13	0.13	0.13	0.13	0.13
Initial temperature, °C	21.4	17.7	17.4	20.2	20.6
Minimal temperature of adsorbent, °C	−22.8	−24.4	−25.0	−24.0	−22.7
Δ*V_a_*, m^3^ STP:					
isothermal charging	3.29	3.44	2.68	4.64	4.88
full charging	5.17	5.38	4.74	6.55	6.92
*τ_e_*, min with *ΔT_e_* = 5 K:					
isothermal charging	13.5	22.5	11.4	13.2	10.1
low-temperature charging	35.6	49.8	48.5	26.7	26.1
*K_he_* with *ΔT_e_* = 5 K	0.74	0.76	0.78	0.81	0.76
